# Family planning utilization among postpartum women in the Bule Hora District, southern Ethiopia

**DOI:** 10.3389/fgwh.2024.1323024

**Published:** 2024-12-09

**Authors:** Nurye Sirage, Zewuditu Desalegn, Wako Golicha Wako, Ali Yimer, Fassikaw Kebede Bizuneh, Sefineh Fenta Feleke, Adem Yesuf, Belda Negesa Beyene

**Affiliations:** ^1^ School of Midwifery, College of Health Sciences, Woldia University, Woldia, Ethiopia; ^2^ Department of Nursing, Bule Hora University Teaching Hospital, Bule Hora, Ethiopia; ^3^ Department of Public Health, Institute of Health, Bule Hora University, Bule Hora, Ethiopia; ^4^ School of Public Health, College of Health Sciences, Woldia University, Woldia, Ethiopia; ^5^ Department of Midwifery, Institute of Health, Bule Hora University, Bule Hora, Ethiopia

**Keywords:** utilization, postpartum, family planning, contraceptive methods, postpartum family planning utilization

## Abstract

**Background:**

Contraception use remains low in Ethiopia, particularly within the first year after childbirth. While some women might have medical conditions that limit their contraceptive options, the primary obstacle to wider family planning adoption is not a specific health problem. Instead, it is the lack of equitable access to high-quality family planning services. This barrier significantly hinders women's ability to make informed decisions about their reproductive health. This study examines postpartum family planning utilization and its associated factors among postpartum mothers in the Bule Hora District.

**Methods:**

We conducted a community-based cross-sectional study. A multistage sampling technique was employed to recruit a total of 630 women who had given birth. To collect the data, structured, standardized, and pretested questionnaires were used, and the collected data were coded and entered into Epi-data version 4.6. The data were analyzed using SPSS version 25. Both bivariable and multivariable logistic regressions were used to identify factors associated with postpartum family planning utilization.

**Results:**

The study found that 71.3% of women utilized postpartum family planning. Significant associations were found between postpartum family planning utilization and various factors, including counseling on family planning during pregnancy [adjusted odds ratio (AOR) = 1.79, 95% confidence interval (CI) 1.61–2.82], delivery (AOR = 2.62, 95% CI 1.56–4.38), and the postpartum period (AOR = 2.71, 95% CI 1.75–4.21). Women who resumed sexual activity after birth (AOR = 1.92, 95% CI 1.25–2.96), and who had at least four antenatal care visits (AOR = 3.09, 95% CI 1.61–5.92) were also more likely to use postpartum family planning. Women with grand multiparity were 69% less likely to use family planning methods than primiparous women (AOR = 0.31, 95% CI 0.13–0.73).

**Conclusion:**

Postpartum family planning use in this study was higher than the national average. Factors such as parity; counseling during the pregnancy, delivery, and postpartum periods; and early resumption of sexual activity were linked to increased contraceptive use. These findings suggest that enhanced counseling during antenatal, delivery, and postnatal care could significantly increase contraceptive use.

## Introduction

Postpartum family planning (PPFP) services are offered to women and couples within the first year after childbirth. These services include counseling and providing contraceptive options. The goal of postpartum family planning is to address the needs of individuals who want to space their children and those who have reached their desired family size (1).

Family planning (FP) helps women, and their partners control the number of children they have and the timing between births. PPFP specifically allows families to prevent unplanned pregnancies in the 12 months following childbirth. It is recommended that after giving birth, a woman should wait at least 2 years before becoming pregnant again to reduce risks to her health, as well as to the health of her baby and pregnancy (2, 3).

Family planning has been one of the most effective development initiatives in the last 50 years. It offers a wide array of benefits, including economic growth, better maternal and child health, advancements in education, and increased empowerment for women. Research indicates that when governments implement high-quality voluntary family planning programs, they can lower fertility rates and achieve significant improvements in health, wealth, human rights, and education (4).

Identifying the various barriers to effective postpartum family planning is crucial. These barriers may include low socioeconomic status, low literacy levels among both partners, young maternal age, non-satisfaction with family planning methods, myths about family planning methods, the need for spousal permission, fears of health issues related to contraception, and negative past experiences with contraceptive methods (5). It is our responsibility to address these challenges through proper counseling and interventions during the antenatal, natal, and postnatal periods, as well as during immunization sessions.

While there are many barriers, there are also significant opportunities to promote family planning. These include access to prior family planning information; the guidance received during health facility visits; antenatal care (ANC); institutional deliveries; skilled birth attendance; counseling on family planning during antenatal, delivery, and postnatal care (PNC); and having a positive attitude (6–8).

Short pregnancy intervals are associated with adverse maternal outcomes, infant mortality, preterm birth, low birth weight, malnutrition, and stunting of children under 5 years, and the majority of these complications require multiple clinic visits and numerous medications and procedures for relief or cure, at a high cost to the healthcare system (9). Providing contraceptive services during the first 12-month period is one of the most cost-effective ways of minimizing avoidable deaths and suffering in women (10).

The World Health Organization recommends a minimum delay of 24 months following a live birth before attempting the next pregnancy to reduce the risk of unfavorable maternal, perinatal, and infant outcomes (3). Birth-to-pregnancy intervals of approximately 18 months or less are linked to increased risks of infant, neonatal, and perinatal mortality, low birth weight, small size for gestational age, and preterm delivery (11).

Many families may disregard contraception after childbirth due to misunderstandings about pregnancy risk, difficulties in accessing services, and cultural concerns (12). PPFP aims to prevent unwanted pregnancies and pregnancies that are too close together following childbirth (13).

In Ethiopia, the utilization rate of postpartum PPFP varies significantly, ranging from 12.9% to 45.8% (8, 14–16). To address this issue and improve family planning services, Ethiopia has launched the Family Planning Costed Implementation Plan for 2023–2030, aiming for a 54% contraceptive prevalence rate by 2030 (17).

The primary goal of the current study is to investigate the utilization of PPFP and identify the associated factors among women who have given birth in the past year. By providing essential data on the prevalence and various factors influencing PPFP use, this study aims to inform and motivate stakeholders including healthcare professionals, administrators, policymakers, and community health promoters to enhance PPFP utilization. The findings are intended to support efforts to increase awareness and accessibility of postpartum family planning services, ultimately contributing to the objectives outlined in the Family Planning Costed Implementation Plan (FP-CIP).

## Methods and materials

### Study design and population

This cross-sectional study was conducted in the Bule Hora District, West Guji Zone, Oromia Region, Ethiopia, from 1 May to 30 June 2021, focusing on women of childbearing age. The zone has nine districts. Of these, only three districts (Suro Barguda, Melka Soda, and Dugda Dawa) are pastoralist areas. The study area has a population of 287,904, including 63,627 women of childbearing age, with approximately 9,990 births in the past year. All women who gave birth in the Bule Hora District within the past year (1 May 2020 to 30 April 2021) were the source population for the study, whereas women who gave birth within the same time frame from the selected kebeles were the study population. Women were included if they had lived in the study area for at least 6 months, while those who were critically ill or unable to communicate during the data collection period were excluded from the study.

### Sample size determination

The sample size was calculated using a single population proportion formula based on the following assumptions: the utilization of postpartum family planning in Ethiopia (45.8%) (18); Zα/2 = the critical value for normal distribution at the 95% confidence level, which is equal to 1.96 (*Z*-value of alpha = 0.05); and a 5% margin of error. The final sample size was 630 after factoring in a 10% non-response rate and 1.5 design effects.

### Sampling procedures and techniques

A multistage sampling technique was used to select the study participants. First, a total of 41 kebeles in the Bule Hora District were identified, with a total of 59,980 households. Then, 12 kebeles were selected via the lottery method. The number of mothers who gave birth in the last 12 months was obtained from the District Health Bureau. On the basis of the information obtained, a proportional size allocation was made for each individual selected according to their population.

In the second sampling stage, a household with a woman who gave birth in the last 12 months was selected by a simple random sampling technique using the prepared list of households as a sample frame and computer-generated numbers. Finally, mothers who gave birth in the extended postpartum period (1 May 2020 to 30 April 2021) were interviewed. Those mothers who were not available on the first visit were revisited three times.

### Study variables

The dependent variable was the utilization of postpartum family planning, whereas age, marital status, educational status of women, educational status of partners, occupation, number of live children, and asset variables, ANC, FP counseling during pregnancy, place of delivery, PNC visit, FP counseling at delivery, counseling during PNC, and birth assistance were all independent variables.

### Measurement

Modern contraceptives include sterilization (male and female), subdermal implants, oral contraceptives, condoms (male and female), injectable emergency contraceptive pills, patches, diaphragms and cervical caps, and spermicidal agents (19).

Utilization of PPFP: When a woman who has recently given birth states that she is using any contemporary birth control methods (such as the pill, intrauterine device, injection, male or female condoms, male or female sterilization, or implants) within the first 12 months following her last childbirth (20).

Menstrual resumption: women who resumed menstruation after giving birth.

Sexual resumption: women who have resumed sexual relationships after giving birth.

Wealth is defined as all natural, physical, and financial assets owned by a household and reduced by its liabilities (21).

Wealth index: A principal component analysis (PCA) was used to create a wealth index score for each household based on the selected indicators, which is calculated by combining the standardized indicators. Households were categorized into wealthy quintiles or categories with each quintile representing 20% of the population. The categories included poorest, poor, middle, rich, and richest.

### Data collection quality and quality control

The data were collected by 12 health extension workers, via structured and pretested questionnaires (22). Four midwives with BSc supervised the overall data collection process. The principal investigators provided 2 days of training for the data collectors before the actual data collection, with a focus on the relevance of the study, the content of the tool, maintaining confidentiality, and data collection procedures.

The questionnaire was first prepared in English, translated into Afan Oromo, and then retranslated back into English to ensure accuracy by language experts. The questionnaires were pretested on 5% of the sample size of the study area (Burka Arbicho Kebele), which has characteristics like those of the study population, to ensure the clarity of the questionnaire. The data included sociodemographic and economic factors, items related to the benefits of using modern contraceptive methods, maternal health service utilization, and the obstetric characteristics of the study subjects. The collected data were checked for completeness and consistency by supervisors and the principal investigator at the end of each day.

### Data processing and analysis

The data accuracy, consistency, and completeness were examined. The data were subsequently entered into Epi-data version 4.6. The data were cleaned and analyzed via SPSS version 25. To describe the characteristics of the study participants, descriptive statistics such as the means, frequencies, and percentages were used. To examine the associations between the dependent and independent variables, a logistic regression was performed. Bivariable analysis was used to select candidate variables for the multivariable model. Variables with a *p*-value of ≤0.25 that were associated with postpartum family planning utilization were entered into the multivariable logistic regression model. The crude and adjusted odds ratios (AOR), as well as 95% confidence intervals (CI), were used to determine the strength of the associations. Before the last multivariable analysis, the logistic regression assumption was verified via the variance inflation factor and was not detected. Model goodness of fit was checked via the Hosmer–Lemeshow test and its value was insignificant (0.35). The statistical significance of the findings of this study was indicated by a *p*-value <0.05. The results are presented in text, tables, and graphs depending on the type of data. The asset variables were calculated via the PCA.

## Results

### Sociodemographic characteristics

This study included 627 women from a total of 630 postpartum mothers in the selected kebeles, with a response rate of 99.5%. The mean age of the respondents was 28.6 (SD ± 5.1) years, with ages ranging from 18 to 47 years. Among the participants, 593 (94.6%) were married women, and 270 (43.1%) were housewives. A total of 451 (71.9%) had no formal education, whereas 13 (2.1%) had a higher level of education (Table 1).

**Table 1:** Sociodemographic and socioeconomic characteristics of mothers in the Bule Hora Woreda, southern Ethiopia, in 2021 (*n* = 627).

Variable	Category	Frequency	Percentage
Age (years)	15–19	13	2.1
	20–24	107	17.1
	25–29	246	39.2
	30–34	164	26.2
	>=35	97	15.5
Marital status	Married	593	94.6
	Unmarried	21	3.3
	Divorce	7	1.1
	Widowed	6	1.0
Level of education	No formal education	451	71.9
	Primary education	131	20.9
	Secondary education	32	5.1
	Tertiary education	13	2.1
Partner’s educational status	No formal education	343	54.7
	Primary education	194	30.9
	Secondary education	66	10.5
	Tertiary education	24	3.8
Wealth index	Poorest	131	20.9
	Poor	118	18.8
	Middle	127	20.3
	Rich	125	20.0
	Richest	125	20.0

### Reproductive and maternal health service-related characteristics

The research involved a majority of women who had given birth multiple times (50.6%), with the highest percentage of participants (45.1%) having received two to three antenatal care visits. A significant proportion (69.7%) received family planning counseling before delivery. A majority (74.6%) experienced unplanned pregnancies, and 69.7% and 66.5% received PPFP information before and after delivery, respectively. While 82.3% received postnatal care, only 66.5% received PPFP counseling during this period. Most users of PPFP (71.3%) received contraceptives from government institutions. The majority of deliveries (71.3%) took place in health institutions, and 72.9% of these deliveries were attended by trained health professionals. The study participants’ reproductive preferences were categorized into five groups. The most common preference was to have another child after 2 years (57.7%, 362). A substantial number also expressed a desire to have another child soon (11.8%, 74). Conversely, 77 (12.3%) reported that they did not want any more children. A smaller proportion (7.5%, 47) were open to having another child at some unspecified point in the future, while 67 (10.7%) remained undecided about their future reproductive plans (Table 2).

**Table 2:** Reproductive and maternal health service-related characteristics in the Bule Hora District, southern Ethiopia (*n* = 627).

Variable	Category	Frequency	Percentage
Parity	Primiparous	56	8.9
	Multiparous	317	50.6
	Grand multiparous	254	40.5
Number of ANC visits	1	74	11.8
	2–3	283	45.1
	≥4	199	31.7
Attend PNC to the last child	Yes	516	82.3
	No	111	17.7
Menses return	Yes	287	45.8
	No	340	54.2
Time of menses return (months)	≤3	187	29.8
	4–6	67	10.3
	7–9	33	5.3
Resumed sexual intercourse	Yes	387	61.7
	No	240	38.3
Time resumed sexual intercourse (weeks)	<6	32	5.1
	6	80	12.8
	≥7	274	43.7
Time started contraceptive methods	Immediately	57	9.1
	At 6 weeks	308	49.1
	After 6 months	132	21.1
Age of recent child (months)	≤3	145	23.1
	4–6	182	29.0
	7–9	133	21.2
	10–12	167	26.6

### Postpartum family planning method utilization by type

**Figure 1:**
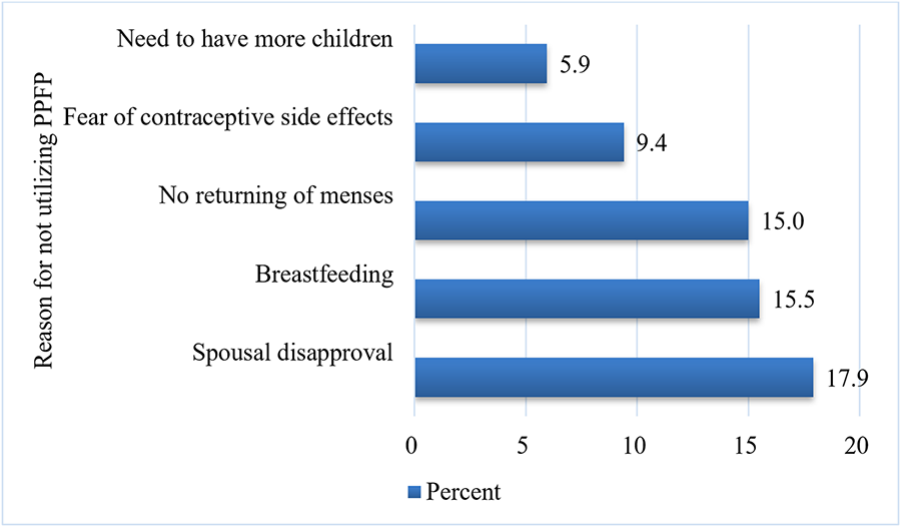
Reason for not utilizing postpartum family planning among women in the Bule Hora District, southern Ethiopia. PPFP, postpartum family planning.

In total, 447 (71.3%; 95% CI 67.6–74.8) respondents had used postpartum contraceptives within the previous year. In this study, injectable contraception was the most commonly used method (47.4%), followed by implants (15.8%). The main reason given by the study participants for not utilizing family planning was spousal disapproval (112, 17.9%) (Figure 1).

### Factors associated with postpartum family planning utilization

In the multivariable logistic regression, receiving counseling about family planning while pregnant, at the time of delivery, and during PNC; resuming sexual activity after birth; planning the last delivery; and the number of ANC visits were significantly associated with postpartum family planning utilization.

Grand multiparous women were 69% less likely to use family planning compared to primiparous women (AOR = 0.31, 95% CI 0.13–0.73).

Women who received family planning counseling during pregnancy were 1.79 times more likely to use family planning than those who did not receive counseling (AOR = 1.79, 95% CI = 1.61–2.82). Women who received family planning counseling at the time of delivery were 2.62 times more likely to use family planning than those who did not receive counseling (AOR = 2.62, 95% CI = 1.57–4.38). Women who received family planning counseling during postnatal care were 2.7 times more likely to use family planning in the postpartum period compared to those who did not receive counseling (AOR = 2.71, 95% CI = 1.75–4.21).

Women who had resumed sexual activity had nearly twice the odds of taking a modern contraceptive as those who had not resumed sexual activity since birth (AOR = 1.92, 95% CI = 1.25–2.96). Mothers who had four or more antenatal care visits were three times more likely to utilize postpartum family planning than those who had fewer visits (AOR = 3.09, 95% CI = 1.61–5.92) (Table 3).

**Table 3:** Bivariable and multivariable logistic regression analyses of factors associated with the utilization of PPFP in the Bule Hora District, southern Ethiopia.

Variable	PPFP utilization		COR (95% CI)	AOR (95% CI)	*p*-value
	No	Yes			
Counseled PPFP at delivery					
Yes	115 (23.1)	383 (76.9)	3.38 (2.26–5.06)	2.62 (1.57–4.38)	0.000^a^
No	65 (50.4)	64 (49.6)	1	1	
Reproductive preference					
I want another child soon	25 (33.8)	49 (66.2)	1	1	
After 2 years	81 (22.4)	281 (77.6)	1.77 (1.03–3.04)	0.619 (0.23–1.65)	0.338
No more child	35 (45.5)	42 (54.5)	0.61 (0.32–1.182)	1.01 (0.45–2.24)	0.984
Want another, but undecided about when to have	18 (38.3)	29 (61.7)	0.82 (0.38–1.76)	0.64 (0.25–1.63)	0.347
Not decided	21 (31.3)	46 (68.7)	1.12 (0.55–2.26)	0.70 (0.26–1.92)	0.491
Menses resumed					
Yes	64 (22.3)	232 (77.7)	1.80 (1.26–2.58)	1.24 (0.77–2.01)	0.380
No	116 (34.1)	224 (65.9)	1	1	
Resumed sexual activity					
Yes	92 (23.8)	295 (76.2)	1.86 (1.31,2.64)	1.92 (1.25–2.96)	0.003^a^
No	88 (36.7)	152 (63.3)	1	1	
Counseled PPFP during pregnancy					
Yes	100 (22.9)	337 (77.1)	2.45 (1.70–3.53)	1.79 (1.61–2.82)	0.012^a^
No	80 (42.1)	110 (57.9)	1	1	
Have PNC visit					
Yes	140 (27.1)	376 (72.9)	1.51 (0.98–2.33)	0.76 (0.41–1.41)	0.387
No	40 (36.0)	71 (64.0)	1	1	
Counseled FP during PNC					
Yes	87 (20.9)	330 (79.1)	3.02 (2.10–4.32)	2.71 (1.75–4.21)	0.000^a^
No	93 (44.3)	117 (55.7)	1	1	
Parity					
1	11 (19.6)	45 (80.4)	1	1	
2–4	70 (22.1)	247 (77.9)	0.86 (0.42–1.76)	0.68 (0.29–1.59)	0.370
≥5	99 (39.0)	155 (61.0)	0.38 (0.19–0.78)	0.31 (0.13–0.73)	0.008^a^
Number of ANC visits					
1	32 (43.2)	32 (43.2)	1	1	
2–3	87 (30.7)	196 (69.3)	1.72 (1.02–2.91)	1.42 (0.79–2.56	0.244
≥4	38 (19.1)	161 (80.9)	3.23 (1.81–5.77)	3.09 (1.10–5.92)	0.001^a^

ANC, antenatal care; AOR, adjusted odds ratio; COR, crude odds ratio; PPFP, postpartum family planning; PNC, postnatal care.

^a^ Significant at *p*-value <0.05.

## Discussion

The total prevalence of postpartum family planning utilization was 71.3%, with a 95% confidence interval of 67.6%–74.8%. The number of antenatal care visits; counseling during pregnancy, delivery, and postnatal care; resuming sexual activity; and anticipated last delivery were significantly associated with postpartum family planning utilization.

The prevalence of PPFP utilization in this study, at 71.3%, aligns with findings from a study in Hossana Town (72.9%) (23), suggesting a consistent trend in certain regions of Ethiopia. However, this rate is lower than that observed in Addis Ababa (80.3%) (12), potentially due to differences in access to healthcare services and sociodemographic factors. The study population in Addis Ababa may have had a higher proportion of women with better educational attainment and residing in urban areas, contributing to higher PPFP utilization.

The prevalence of PPFP utilization observed in this study (71.3%) is also significantly higher than that reported in previous studies conducted in Gondor (45.8%) (24) and Debre Birhan, Ethiopia (33.1%) (25). The difference could be due to differences in the reproductive and maternal health service use-related characteristics of the study participants.

Women who had received family planning counseling during postnatal care follow-ups were more likely to use postpartum contraceptives than those who did not. This finding was supported by studies undertaken in northern Ethiopia (12), the Aroressa District (26), Kebribeyah Town, Ethiopia (27), and the Kailali district in Nepal (28). PNC counseling plays a crucial role in promoting PPFP utilization. By introducing the concept of family planning during PNC visits, healthcare providers create an opportunity for women to reflect on their future family planning needs. This early engagement allows women to address concerns, understand the benefits of spacing pregnancies, and explore their options. Comprehensive PNC counseling also increases women's awareness of different contraceptive methods, their effectiveness, and access points. This knowledge empowers them to make informed choices about family planning. Furthermore, a supportive and respectful PNC environment fosters trust between women and healthcare providers. This trust is essential for open conversations about family planning, encouraging women to feel comfortable discussing their needs and seeking PPFP counseling.

Women who were counseled about postpartum contraception at the time of birth had a statistically significant association with postpartum family planning utilization. This finding is in line with a study conducted in Sidama, southern Ethiopia (29), and Pawe, Benishangul Gumuz, Ethiopia (30). Counseling during childbirth provides a critical opportunity to introduce PPFP, increasing awareness and acceptance of family planning. This early engagement builds trust and empowers women to discuss their future family planning needs, leading to higher PPFP utilization rates. This highlights the importance of integrating PPFP counseling into childbirth care as a standard practice. Policymakers should prioritize training healthcare providers to effectively deliver this counseling and ensure access to a variety of contraceptive methods in all birth settings.

This study suggests a strong link between resuming sexual activity and PPFP utilization, consistent with findings in Ghana (31) and Nigeria (32). Women who have resumed sexual activity are more likely to seek family planning to prevent unplanned pregnancies. Clinicians should tailor counseling strategies to address the needs of women who have resumed sexual activity, while policymakers should strengthen programs to ensure access to culturally appropriate family planning services in all regions. This includes promoting awareness, improving contraceptive access, and training healthcare providers. These efforts empower women to make informed choices about their reproductive health.

This study found that women who completed four or more ANC visits during pregnancy were more likely to utilize PPFP. This finding aligns with research conducted in Kebribeyah Town, Somalia, Eastern Tigray, and Western Ethiopia (27, 33, 34), suggesting a consistent association between increased ANC engagement and PPFP uptake. The likely reason behind this association is that frequent ANC visits provide opportunities for women to receive comprehensive health information, including counseling on family planning and the benefits of spacing pregnancies. Women who attend more ANC visits are more likely to be aware of the risks of closely spaced pregnancies for mothers and newborns, making them more receptive to PPFP.

This finding underscores the importance of integrating family planning counseling into routine ANC visits. Clinicians should leverage these visits to discuss family planning options, address concerns, and provide comprehensive information about spacing pregnancies and their benefits. This proactive approach can significantly improve PPFP utilization.

In this research, it was observed that the adoption of postpartum family planning was lower among women with five or more children compared to those with four or fewer children. Aligning with existing literature, the study revealed that women with four or more children tended to use contraception at notably lower levels than women with one to three children. In addition, married women with four or more births exhibited lower rates of modern contraceptive usage (27.1%) in contrast to women with zero to one child (41.6%) or two to three children (43.6%) (35, 36). This might be due to access barriers and cultural and societal norms, and multiparous women may believe that they have a lower chance of unplanned pregnancies due to natural declining fertility with advanced age.

### Strengths and limitations of the study

The large sample size enhances the study's statistical power and the reliability of the findings. The study examined various factors that positively influence PPFP utilization, providing valuable insights into the determinants of PPFP uptake. Because of the cross-sectional nature of the study, it is not possible to establish a causal relationship between the factors that were identified and the use of postpartum family planning.

## Conclusion

In this study, the prevalence of postpartum family planning utilization was relatively high compared with that reported in previous national studies. Counseling family planning during the pregnancy, delivery, and postnatal periods; resuming sexual activity after birth; multiparity; and the number of antenatal care visits were significantly associated with postpartum family planning utilization.

### Implications for policies and practice

According to the study's findings, policymakers should prioritize training healthcare providers in effective family planning, ensure that all women have access to these services, and emphasize the significance of comprehensive family planning counseling from the beginning of pregnancy to postnatal care.

It is highly recommended that the many misconceptions of PPFP are addressed and the advantages of family planning and its integration with ANC services are highlighted. During the pregnancy, delivery, and postpartum periods, proactive implementation of comprehensive and inclusive counseling is required. A key component is empowering all women to make knowledgeable decisions regarding their reproductive health.

To be able to gain an understanding of the factors driving PPFP utilization and investigate the experiences and viewpoints of women with various parities, future research incorporating qualitative investigations is advised.

## Data Availability

The original contributions presented in the study are included in the article/Supplementary Material, further inquiries can be directed to the corresponding author.
